# The Incidence and Management of Type 2 Diabetes Mellitus After Gestational Diabetes Mellitus

**DOI:** 10.7759/cureus.44468

**Published:** 2023-08-31

**Authors:** Chinyere L Ikoh Rph., Roland Tang Tinong

**Affiliations:** 1 Endocrinology, Diabetes and Metabolism, John F. Kennedy University of Medicine Curacao, Willemstad, CUW; 2 School of Medicine, Caribbean Medical University, Willemstad, CUW

**Keywords:** breastfeeding, postpartum care, lifestyle interventions, risk factors, management, incidence, type 2 diabetes mellitus, gestational diabetes mellitus

## Abstract

Gestational diabetes mellitus (GDM) refers to a transient state of impaired glucose tolerance that develops during pregnancy, affecting a significant proportion of expectant mothers globally. This review aimed to comprehensively examine the subsequent incidence and management of type 2 diabetes mellitus (T2DM) in women who have previously experienced GDM. The transition from GDM to T2DM is a well-recognized continuum, with affected women facing an increased risk of developing T2DM postpartum. Several studies have demonstrated that women with a history of GDM face a substantially higher risk of developing T2DM compared to normoglycemic pregnant women. The long-term consequences of developing T2DM following GDM are significant, as it not only affects the health of the mother but also poses risks to the offspring. The most common risk factors associated with the progression of GDM to T2DM include pregnancy at an advanced age, insulin treatment during pregnancy, and delivering an overweight baby. As GDM women are at higher risk of developing T2DM, effective management strategies such as lifestyle changes, postpartum care, breastfeeding, screening tests, and gaining awareness of risk are crucial to mitigate the risk of T2DM in this population. The current review was conducted to guide healthcare providers and women with a history of GDM about the potential risks of T2DM and management strategies to prevent the condition. This review provides a summary of evidence on the incidence rate of T2DM in GDM patients, its associated risk factors, and approaches to mitigate this challenge.

## Introduction and background

Gestational diabetes mellitus (GDM) is a condition characterized by hyperglycemia during the second or third trimester of pregnancy in women who have not been diagnosed with diabetes previously [1]. In most cases, this elevation in blood sugar level occurs due to the dysfunction of pancreatic β-cells, thereby compromising glucose tolerance. Advanced maternal age, being overweight, and a family history of diabetes are some of the risk factors for GDM [2]. However, it is important to understand that hyperglycemia identified during pregnancy is not always due to GDM, and it could be due to previously existing type 2 diabetes mellitus (T2DM) that had remained undiagnosed. Mothers suffering from GDM and their offspring are at higher risk of developing serious health consequences later in life [3,4]. One of the post-GDM complications is T2DM. Several studies have suggested that women suffering from GDM during the first nine months of pregnancy carry a 4-10% risk of developing T2DM [5-8]. A meta-analysis of 20 studies involving 1,332,373 participants (67,956 GDM women) by Li et al. reported that the risk of T2DM is 10-fold higher in women who suffer from GDM compared to normoglycemic pregnant women [9]. Based on the available evidence, a diagnosis of GDM can uncover the risk of T2DM in such patients. The susceptibility of GDM patients to develop T2DM is not surprising as both of these conditions share the same risk factors such as increased BMI [10]. Hence, the continuum from GDM to T2DM presents a unique opportunity for early intervention and implementing preventive strategies to mitigate the long-term burden of metabolic disorders in affected individuals.

Despite the magnitude of risk associated with GDM, the postpartum screening of T2DM remains suboptimal [11]. A retrospective study by Goueslard et al. reported that less than 25% of women with GDM undergo T2DM screening within the first three months of delivery whereas less than 60% of women get screened for T2DM within one year in France [12]. This shows a general lack of awareness among GDM women about the increased risk of T2DM. Furthermore, healthcare providers are also not adequately informed about screening guidelines [13]. Several systemic reviews and meta-analyses have reported the incidence rate of T2DM in GDM patients. For instance, a systemic review and meta-analysis by Kim et al. reported an incidence of T2DM between 2.6-70%, with the highest incidence observable within five years after delivery [14]. In another systemic review and meta-analysis, Bellamy et al. found a seven-fold increased incidence of T2DM after the diagnosis of GDM [3]. Another systemic review focusing on Asian women reported that the incidence of T2DM after GDM is 58%, with only 2.8% of women undergoing screening after delivery [15]. Despite the substantial evidence about the incidence of T2DM after GDM, there is still a lack of studies assessing both incidences and recommended managemental strategies. Given the rise in obesity across the world and the more advanced age of pregnancy, there is a need to raise awareness among the general public and healthcare providers regarding the risk of GDM and subsequent T2DM and the ways to mitigate this risk. In light of this, this review aims to provide a summary of findings of about the diagnosis, risk factors, and management of T2DM after GDM. To understand the progression of T2DM from GDM, it is important to understand how GDM and T2DM affect women and how they can be effectively screened.

## Review

Gestational diabetes mellitus (GDM)

The first case of GDM was described in 1824 by Bennewitz in Germany [16]. Regarding the incidence of GDM, any pregnant woman who never had any kind of diabetes mellitus can acquire it during pregnancy. In fact, GDM is not a disease, rather it is a temporary pregnancy-related complication. In this condition, the hormonal system of the mother is unable to retain normal sugar levels in the blood [17]. It is a very common pregnancy-related complication and most of the women with GDM go on to give birth to healthy babies. GDM women rarely show any symptoms; however, excessive thirst and increased urination can occur in some cases [18]. Like other types of diabetes, GDM involves the dysfunction of pancreatic β-cells. According to available evidence, defects in β-cells during GDM result from the same factors that cause diabetes in general, such as insulin resistance, autoimmune disease, and monogenic causes [5]. Gestational diabetes can be fully treated but careful supervision is required throughout the pregnancy [19]. If left untreated, GDM can cause poor fetal and maternal outcomes: stillbirth is more likely to occur in women with GDM [20]. Moreover, there is an increased risk of macrosomia, preterm delivery, and large gestational age in infants, causing respiratory distress, neonatal hypoglycemia, and birth injury [21]. Furthermore, children of mothers with gestational diabetes mellitus have a greater risk of developing T2DM and obesity later in life [22-23]. Normal glucose level is achieved after delivery in most cases but the risk of developing T2DM in the future remains higher in these patients. According to the literature, the rates at which GDM develops into T2DM vary and range from 3% to 70% [24-27]. This variation in development rates can be attributed to genetic factors, variation in follow-up duration, biases in the selection of participants in the studies, and variation in tests to measure glucose tolerance in women during pregnancy [26]. Furthermore, the risk of GDM recurrence is higher in subsequent pregnancies among these women. A study by Getahun et al. reported that the risk of GDM is 41.3% and 4.2% in the second pregnancy following a GDM-positive and negative first pregnancy, respectively [28]. Since the risk of GDM recurrence increases, it is plausible that the risk of T2DM will also increase in multiple affected pregnancies.

Pathophysiology of gestational diabetes mellitus

**Figure 1:**
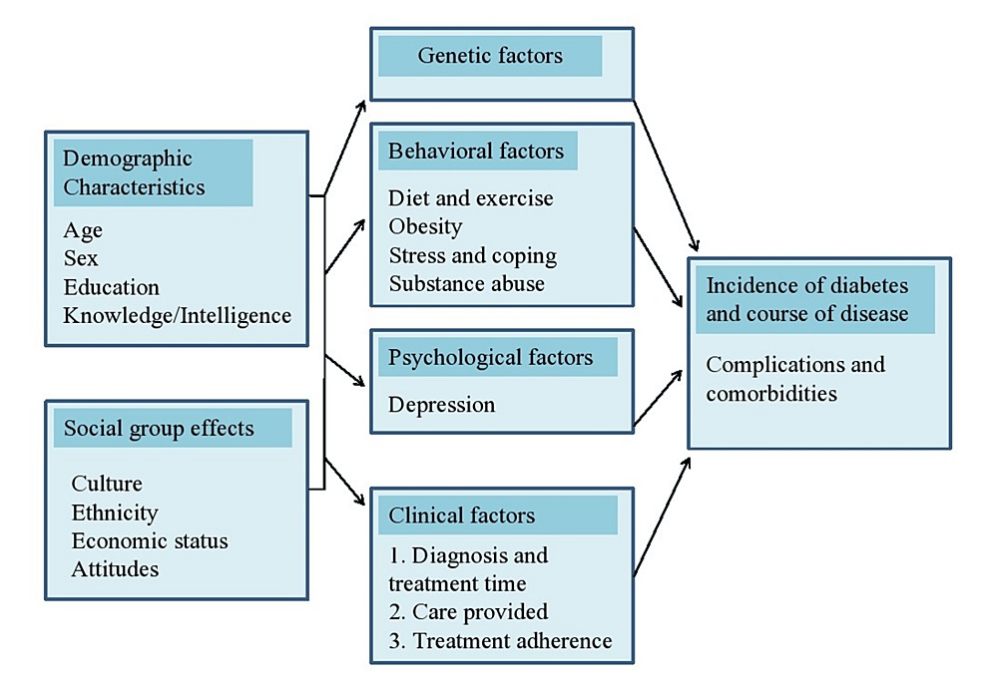
Probable factors contributing to the development of gestational diabetes mellitus in individuals with genetic vulnerability

The pathophysiology of GDM involves an intricate interplay between hormonal, genetic, and environmental factors (Figure 1). During pregnancy, the concentration of pregnancy-related hormones (progestins and estrogens) increases. This rise in concentration has a number of consequences including slow gastric emptying, and low concentration of fasting glucose. With the progression of gestation, tissues become less sensitive to insulin and the concentration of postprandial glucose increases as a result [29]. In pregnant women, insulin should be secreted in an adequate concentration by pancreatic β-cells to cancel out the consequent fall in sensitivity of tissues to insulin. Women suffering from GDM fail to secrete enough insulin to recompense their resistance to insulin [30]. In pregnant women having GDM, the insulin receptor B is unable to undertake tyrosine phosphorylation [31].

Testing for and diagnosis of gestational diabetes mellitus

Screening for gestational diabetes GDM is usually done at 24-28 weeks after the start of pregnancy. During the second trimester, insulin resistance becomes higher, and levels of glucose increase in women who are unable to produce an adequate amount of insulin [32].

The World Health Organization (WHO) put in place the following criteria for GDM diagnosis in 1999:

1. In the first trimester and at the start of the second trimester, the concentrations of postprandial and fasting glucose are less than those in non-pregnant women. A rise in postprandial and fasting glucose concentrations at this stage of pregnancy can reflect the occurrence of GDM.

2. GDM is tested at 24-28 weeks of pregnancy.

3. To find out the presence of GDM, a standard oral glucose tolerance (OGTT) test is carried out with 75 g of anhydrous glucose in 250-300 ml of water after fasting for about 8-14 hours. Glucose levels in plasma are determined in fasting conditions and after two hours of breaking the fast. During pregnancy, women meeting the criteria of diabetes mellitus are said to have GDM [33]. Table 1 summarizes the criteria for diagnosing GDM by various organizations.

**Table 1:** Criteria to diagnose gestational diabetes mellitus WHO: World Health Organization; ACOG: American College of Obstetricians and Gynecologists; CDA: Canadian Diabetes Association; IADPSG: International Association of Diabetes and Pregnancy Study Groups; DIPSI: Diabetes in Pregnancy Study Group of India; PG: plasma glucose; OGTT: Oral glucose tolerance tests

Guidelines	Fasting PG, mg/dl (mmol/l)	Glucose challenge	1-hour PG, mg/dl (mmol/l)	2-hour PG, mg/dl (mmol/l)	3-hour PG, mg/dl (mmol/l)
WHO 1999	≥126 (7.0)	75 g OGTT	Not required	≥140 (7.8)	Not required
CDA	≥95 (5.3)	75 g OGTT	≥191 (10.6)	≥160 (8.9)	Not required
DIPSI	Not required	75 g OGTT	Not required	≥140 (7.8)	Not required
ACOG	≥95 (5.3)	100 g OGTT	≥180 (10.0)	≥155 (8.6)	≥140 (7.8)
IADPSG	≥92 (5.1)	75 g OGTT	≥180 (10.0)	≥153 (8.5)	Not required

Type 2 diabetes mellitus (T2DM)

T2DM occurs due to the impairment regarding resistance to insulin, secretion of insulin, or both. T2DM is more widespread than GDM and T1DM and accounts for more than 90% of all diabetic cases. The understanding of how this disease develops and progresses has evolved rapidly over the last few decades. The major cause of T2DM is impairment in the production of insulin by β‑cells of the pancreas. Overt hyperglycemia turns into prediabetes [34,35]. Prediabetes is a risk factor for T2DM. Prediabetes is characterized by symptoms including impaired glucose tolerance (IGT), increased levels of HbA1c, and impaired fasting glucose (IFG) levels.

Patients having IGF levels have fasting plasma glucose (FPG) levels higher than normal but lower than diabetic levels. IGT involves the resistance of insulin in muscles and the late secretion of insulin after a meal. Levels of HbA1c in prediabetic individuals range from 5.7 to 6.4%. The annual rates at which prediabetes converts to diabetes have been reported to be 3-11% [36].

Pathophysiology of type 2 diabetes mellitus

**Figure 2:**
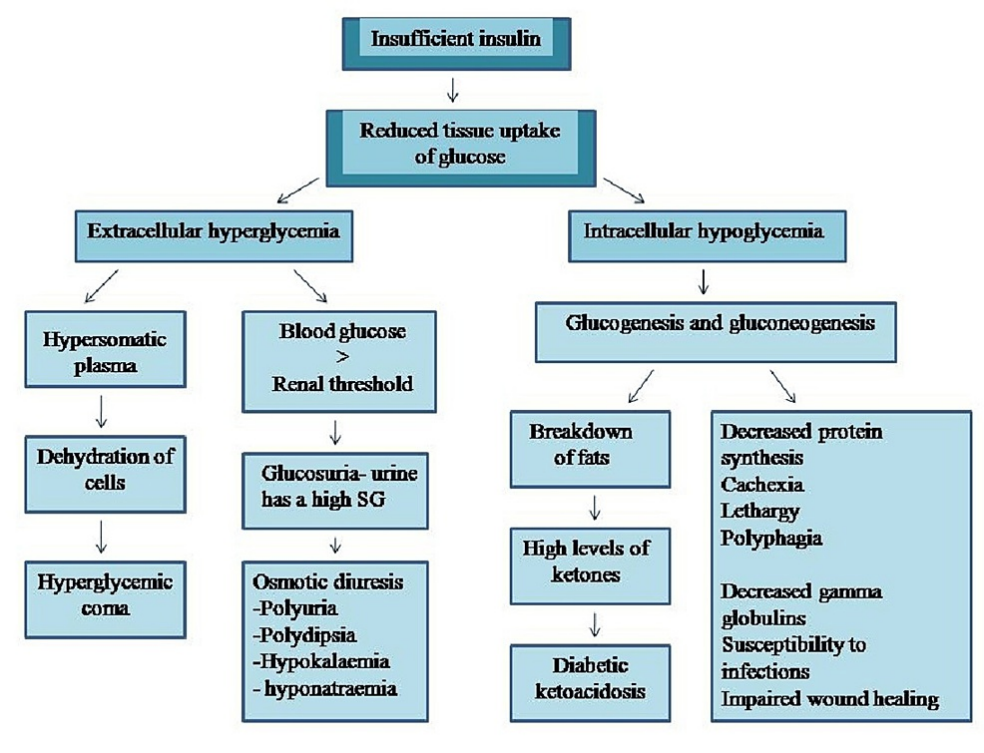
Effects of insufficient insulin on the body

In T2DM, glucose levels in the blood become high due to improper functioning of feedback loops among insulin secretion and its action [37] (Figure 2). Due to improper working of β-cell, a reduction in the secretion of insulin occurs and the body fails to maintain normal levels of glucose in blood. Moreover, the liver produces more insulin and a reduction in glucose uptake by muscles occurs due to resistance to insulin. When resistance to insulin, as well as β-cell dysfunction, are present, an amplification in hyperglycemia occurs, resulting in T2DM [38-39].

Screening for and diagnosis of type 2 diabetes mellitus

Even though T2DM is a very common medical condition and a number of tests are available for its screening and diagnosis, it is still underdiagnosed. Around 25% of individuals with newly diagnosed diabetes already suffer from microvascular disease. It indicates that they have had T2DM for four to seven years before its diagnosis [40-41]. For the diagnosis of T2DM, specific tests are performed to determine the hyperglycemia level. The FPG test is used to determine plasma glucose and is performed after at least eight hours of fasting. This test is preferred because it is risk-free, easy to perform, and inexpensive [41]. Another test for T2DM diagnosis is the random plasma glucose (RPG) test. This test may be performed at any time because fasting is not required for it [42]. The OGTT was first used in 1922 and has been used since then for T2DM screening [43]. This test is used to formally diagnose impaired glucose tolerance. This test diagnoses around 2% more diabetes cases than the FPG test [44].

Incidence of type 2 diabetes mellitus after gestational diabetes mellitus

Globally, T2DM is becoming a serious health problem among a wide range of adult men and women [45-48]. In pregnant women, GDM poses a significant concern and it can have various health consequences later in life. Therefore, it is important to diagnose it as soon as possible to prevent the high risk of chronic diabetes [49]. The primary factors contributing to GDM in pregnant women are as follows: advanced age and higher BMI [50-51], low insulin levels, genetic history, increased invasion of parasites, and disabled function of pancreatic β-cell [7,26,52-54]. Early diagnosis is crucial, and regular glucose screening tests at short intervals are recommended to identify the disease in a timely manner [55-56]. Unfortunately, GDM often receives insufficient attention from obstetricians, which increases the risk of T2DM among women after giving birth [3] (Table 2).

**Table 2:** Risk factors associated with the transition of GDM to T2DM BMI: body mass index; GDM: gestational diabetes mellitus; T2DM: type 2 diabetes mellitus

General factors	Factors related to pregnancy
Waist circumference	Gestational diabetes mellitus
BMI	Using Insulin for gestational diabetes mellitus
Fasting glucose level	Breastfeeding for a short duration
Family history of diabetes	Gestational weight gain
Age	

Materials and methods

For performing this review, a comprehensive search was conducted on PubMed, Scopus, and CINAHL databases to find relevant studies. The search involved a combination of different keywords, including “type 2 diabetes mellitus,” “T2DM,” “non-insulin dependent diabetes,” “gestational diabetes,” and “pregnancy-induced diabetes.” Related terms, alternatives, and plurals were also considered. Keywords were searched for individually as well as in combination. Furthermore, we searched Google Scholar and the reference section of the selected studies to enhance the body of evidence in the current review. Studies that fulfilled the following criteria were included: (1) observed the incidence of T2DM in patients with a known history of GDM, (2) focused on the management of T2DM after GDM, and (3) published in English. Furthermore, we confined the search to the period from 2010 to 2023 to provide more recent evidence on the topic.

Results and discussion

Existing literature has demonstrated that T2DM is more likely to develop in patients with a history of GDM (Table 3). However, the incidence rate reported in studies usually varies across ethnic groups and regions across the world. This variation can be attributed to deviations in the screening procedure and in follow-up duration [57]. A study conducted in KSA reported that increased age, second pregnancy, previous multiple childbirths, and genetic diabetic history increased the risks of T2DM [58]. It also reported that 67% of women were found to have developed T2DM on follow-up screening. Apart from developing T2DM, several other health consequences have been reported in GDM patients. For example, Aziz et al. reported that GDM individuals were older and had higher BMI after two years post-delivery. Furthermore, they also reported that babies born to GDM mothers had significantly higher birth weights (3.8 ± 0.5 kg vs. 2.6 ± 0.63) and two-year body weights (10.7 ± 2.3 kg vs. 7.1 ± 1.4 kg) (p<0.05) compared to babies born to non-GDM mothers [59]. Factors that determine the risk of developing T2DM in GDM patients are of significant importance because they can guide future management efforts. Among GDM women, the number of OGTT and insulin use have been linked to a substantially higher risk of T2DM [60]. Similarly, various risk factors have been identified in a previous systemic review involving 129 studies. Among women with a history of GDM occurring for the third time within a span of 15 years, there was a notable increase in the proportion of women diagnosed with T2DM following childbirth. Specifically, for each year after delivery, the incidence of T2DM increased by 12%, with a 95% confidence interval ranging from 8% to 16%. In comparison to other ethnic groups, the occurrence of T2DM in White European populations was found to be lower, accounting for approximately 57% (range: 39-70%). Additionally, for each unit increase in BMI during follow-up, there was an observed increase of 18% (range: 5-34%) in the incidence of T2DM among these populations [61]. Similarly, other studies have shown that geographic distribution pattern, age span, diagnostic standard, and BMI have an influence on the occurrence rate of T2DM in pregnancy, specifically in women with GDM [62]. A study by Herath et al., with a follow-up period of more than 10 years, reported that giving birth after 30 years, insulin treatment during pregnancy, and delivering a baby above 3.5 kg were risk factors for T2DM. They also found that GDM women were 10.6 times more likely to develop T2DM within 10 years compared to non-GDM women [63]. Huopio et al. revealed that increased waist circumference and body weight are significant predictors of the development of T2DM in GDM patients (p<0.001) [64].

**Table 3:** Summary of studies that investigated the incidence of T2DM following GDM GDM: gestational diabetes mellitus; T2DM: type 2 diabetes mellitus

Authors, year of study	Study design	Country	Follow-up (n)	Follow-up (years)	Mean age (years)	Findings
Chodick et al., 2010 [60]	Retrospective cohort	Israel	185,416 (GDM: 11,270, controls: 174,146)	5.4	GDM: 32.74, controls: 30.59	The risk of diabetes development at 10 years was 15.7% vs. 1% in GDM and non-GDM participants
Retnakaran et al., 2010 [65]	Prospective cohort	Canada	180 (GDM: 107, controls: 73)	1	GDM: 35.2, controls: 35.6	The incidence of T2DM in GDM was 34.6% at 3 months and 32.7% at 12 months
Wang et al., 2012 [66]	Prospective cohort	United States	19,998 (GDM: 1,142, controls: 18,856)	8.6	GDM: 26.8, controls: 24.3	The HR of T2DM was 6.52 in GDM and non-GDM women
Mukerji et al., 2012 [67]	Population-based	Canada	1,050,108 (GDM: 33,203, controls: 1,016,905)	15	—	16.5% of GDM Chinese women developed T2DM
Huopio et al., 2014 [64]	Follow-up study	Finland	874 (GDM: 489, controls: 385)	7.3	GDM: 37.8, controls: 38.4	The HR related to the risk of prediabetes and T2DM was 3.7 and 40.7, respectively, in GDM women
Pintaudi et al., 2015 [68]	Population-based	Italy	15,404 (GDM: 3,851, controls: 11,553)	8	GDM and controls: 35.7	54/1,000 GDM women developed T2DM compared to 2.1/1000 non-GDM women
Vigneault et al., 2015 [69]	Prospective cohort	Canada	299 (GDM: 216, controls: 83)	4	GDM: 36.36, controls: 35.66	GDM-positive women with obesity had a T2DM incidence of 21.7% and a prediabetes rate of 72.1%
Minooee et al., 2017 [70]	Population-based	Iran	2,458 (GDM: 476, controls: 1,982)	15	GDM: 36.5, controls: 34.3	In the GDM group, 9/1,000 women developed T2DM compared to 4/1000 in the non-GDM group
Herath et al., 2017 [63]	Retrospective cohort	Sri Lanka	359 (GDM: 119, controls: 240)	10	GDM: 42.7, controls: 38.7	GDM women are 10.6 times more likely to develop T2DM than non-GDM women
Aziz et al., 2018 [59]	Follow-up study	Pakistan	167 (GDM: 78, controls: 89)	2	GDM: 28.9, controls: 25.68	At the 2-year follow-up, 14.1% developed T2DM. Weight at birth and at 2 years was higher in GDM compared to non-GDM (p<0.05)
Daly et al., 2018 [71]	Retrospective cohort	UK	46,399 (GDM: 9,118, controls: 37,281)	25	GDM and controls: 33.0	GDM-positive women were at higher risk of T2DM (IRR: 21.96)
Mahzari et al., 2018 [58]	Retrospective chart review	Saudi Arabia	123 GDM	4	34 ± 4.7	67% of GDM patients developed T2DM
Yefet et al., 2019 [72]	Retrospective cohort	Israel	798 (GDM: 446, controls: 352)	15.8	GDM and controls: 45.0	GDM women who developed T2DM - control: 5.4%, GDM with good glycemic control: 38%, and GDM with poor glycemic control: 57%

Interventions to prevent type 2 diabetes mellitus after gestational diabetes mellitus

Postpartum Care

There are several mitigating strategies that could be followed after the diagnosis of GDM to avoid progression toward T2DM. Optimal care for women affected by GDM requires a proficient healthcare practitioner who can effectively manage the well-being of pregnant individuals throughout the entire gestation period. Due to the multifaceted nature of this condition, it is imperative to involve various disciplines in its management. Furthermore, fostering collaboration among healthcare professionals, raising awareness about health-related matters, and implementing appropriate measures to mitigate the impact of GDM in affected women are crucial components that must be ensured [73]. However, this practice must be maintained in the antenatal period and postnatal as well as in the preconception period. As mentioned in the Australasian Diabetes in Pregnancy Society (ADIPS) guidelines, general practitioners should work with GDM patients to arrange follow-up care, with an OGTT for 6-12 weeks after delivery, a diagnosis of T2DM based on WHO criteria. Subsequently, it is advised that these individuals undergo OGTTs at least twice a year as part of ongoing monitoring and to ascertain the presence of T2DM [74], typically from three to 12 months postpartum depending on levels of dysglycemia if any.

Role of Midwives

The role of midwives is very important to reduce the risk of T2DM in GDM women. It is mandatory to hire midwives for the care of newborn children, which includes breastfeeding and proper testing of affected women [75-77]. Improper maternity and baby care has not only negative implications for GDM women but also long-term economic effects if it develops into T2DM [78]. There could be several barriers to ensuring postpartum prevention of GDM in affected women, including tiredness, lack of childcare options, and work requirements [79]. In such a scenario, the role of midwives becomes even more important to ensure the care of the baby and mother and provide adequate advice regarding the risk of T2DM.

Role of Breastfeeding

Breast milk is a rich source of nutrients for infants. A significant amount of research has highlighted the role of breastfeeding in reducing the risk of T2DM in mothers. When GDM mothers breastfeed, it leads to increased production of serotonin. This increase in serotonin stimulates the multiplication of beta cells and helps in reducing oxidative stress within the pancreas [80]. Thus, breastfeeding is not only beneficial in reducing blood sugar levels due to a decrease in insulin resistance, but it also offers long-term benefits such as preventing T2DM progression, childhood obesity in infants, and ensuring a lower risk of cardiovascular disease [81]. However, breastfeeding rates in GDM mothers are usually lower compared to healthy mothers due to physical discomfort caused by delayed development of mammary glands and difficulties during labor, known as dystocia [82]. Nguyen et al. have reported that GDM is associated with a shorter duration of breastfeeding [83]. Therefore, such women require constant support after hospital discharge to carry on with breastfeeding. In such a scenario, midwives can be helpful in encouraging and supporting breastfeeding. In general, women with elevated BMI and those who are suffering from GDM can benefit from breastfeeding in terms of preventing T2DM [84-85]. The advantages of breastfeeding are dependent on the duration and extent of lactation, as lactation lasting longer increases insulin sensitivity as well as glucose metabolism and lowers the risk of T2DM [85].

Oral Glucose Tolerance Test Screening and Healthy Lifestyle

OGTT screening test is the best diagnostic option to eliminate the risk of T2DM prior to pregnancy [86,87]. Women with high obesity are particularly vulnerable to developing T2DM. To mitigate this risk to a certain extent, it is highly recommended to promote healthy lifestyles and implement awareness programs at national and international levels [88-89]. This disease is a chronic medical condition that can affect both mother and fetus, leading to an increase in body weight [90]. It can also cause changes in the glucose metabolism in developing babies. To avoid these health issues in the future, midwives' care and proper follow-up throughout the whole family must be a concern [91]. In addition, awareness programs must be conducted to educate GDM women, which can be effective in bringing positive change in their lifestyle [92].

Raising Awareness of Risk

Although T2DM is found to develop very commonly after GDM, women during pregnancy are still unaware of its risk, which increases the chances of its development. To increase awareness regarding the risk of developing T2DM after GDM, proper interventions are required. Patient risk awareness can be increased by initiating campaigns for public education. Conferences should be held to demonstrate the factors that can cause GDM. Online information should be made available for individuals about the health risks faced by pregnant women with GDM [93].

Treatment of gestational diabetes mellitus

Very limited data is available regarding the best treatment for controlling glucose levels in the blood. In most instances, the first choice for the treatment of GDM is medical therapy along with weight management or physical activity. A registered dietitian can help by developing a diet plan to control blood sugar levels [94-95]. Furthermore, oral therapy or insulin is clinically used to manage GDM. The decision to use a particular agent for initializing treatment depends on the patient [96]. In individuals with newly diagnosed diabetes mellitus, it is not easy to estimate the levels of insulin production and insulin resistance. Approximate insulin demands in pregnancy are also very difficult to ascertain since resistance to insulin increases with time because of shifts in pregnancy hormones with each trimester. The second-line treatment for GDM usually involves metformin [94-97]. Metformin is more affordable and easier to administer when compared to insulin [98].

Treatment of type 2 diabetes mellitus

Lifestyle changes along with medical treatment are required to control diabetes (Table 4). Maintaining the glucose levels in a normal range helps to decrease the risk of microvascular as well as macrovascular complications. This disease can be treated by administering medication both orally and by injection. In addition to medication, lifestyle changes have also been observed to be helpful in controlling diabetes. Healthcare providers must be aware of various kinds of existing medications available to treat diabetes and should choose the safest, most useful, and easy-to-tolerate drugs for the patients. The first choice to treat T2DM is usually metformin. Other medications are used according to the individual characteristics of the patient, such as allergies [99].

**Table 4:** Treatment options for type 2 diabetes mellitus GLP-1: glucagon-like peptide I; DPP-4: dipeptidyl peptidase 4; SGLT2: sodium-glucose cotransporter-2

Class of drug	Effect	Weight change	Hypoglycemia	Comments
Metformin	Insulin sensitizer	Neutral/loss	No	Gastrointestinal side effects, deficiency of B12, Low eGFR, dehydration, lactic acidosis, hypoxia
Sulphonylurea	Insulin provider	Increase	Yes	Allergy, weight gain, risk for hypoglycemia
Meglitinides	Insulin provider	Increase	Yes	Risk for hypoglycemia
Alfa-glucosidase inhibitor	Glucose absorption inhibitor	Neutral	No	Frequent dosing, gastrointestinal side effects
Pioglitazone	Insulin sensitizer	Increase	No	Heart failure, fractures, edema, urinary bladder cancer
GLP-I agonist	Insulin provider	Decrease	No	Pancreatitis, injectable, gastrointestinal side effects
DPP-4 inhibitor	Insulin provider	Neutral	No	Pancreatitis
Insulin	Insulin provider	Increase	Yes	Risk for hypoglycemia, weight gain, Injectable
SGLT2 inhibitors	Blocks renal glucose absorption in the proximal tubule	Decrease	No	Urinary tract infections

## Conclusions

The incidence of T2DM following GDM poses a significant long-term health concern for women who experience GDM during pregnancy. The available evidence suggests that women with a history of GDM are at a higher risk of developing T2DM compared to women with normoglycemic pregnancies. The most common risk factors associated with the progression of GDM to T2DM include giving birth after 30 years, insulin treatment during pregnancy, and delivering a baby weighing above 3.5 kg. However, proper screening and timely identification can reduce this risk to a great extent. Additionally, certain interventions such as good postpartum care, healthy lifestyle, breastfeeding, and awareness of risk among pregnant women can also be helpful in preventing T2DM after GDM.

## References

[1] (2020). 2. Classification and diagnosis of diabetes: standards of medical care in diabetes-2020. Diabetes Care.

[2] Plows JF, Stanley JL, Baker PN, Reynolds CM, Vickers MH (2018). The pathophysiology of gestational diabetes mellitus. Int J Mol Sci.

[3] Bellamy L, Casas JP, Hingorani AD, Williams D (2009). Type 2 diabetes mellitus after gestational diabetes: a systematic review and meta-analysis. Lancet.

[4] Metzger BE, Coustan DR (1998). Summary and recommendations of the Fourth International Workshop-Conference on Gestational Diabetes Mellitus. The Organizing Committee. Diabetes Care.

[5] Buchanan TA, Xiang A, Kjos SL, Watanabe R (2007). What is gestational diabetes?. Diabetes Care.

[6] Kim C, Berger DK, Chamany S (2007). Recurrence of gestational diabetes mellitus: a systematic review. Diabetes Care.

[7] Feig DS, Zinman B, Wang X, Hux JE (2008). Risk of development of diabetes mellitus after diagnosis of gestational diabetes. CMAJ.

[8] Carr DB, Utzschneider KM, Hull RL (2006). Gestational diabetes mellitus increases the risk of cardiovascular disease in women with a family history of type 2 diabetes. Diabetes Care.

[9] Li Z, Cheng Y, Wang D, Chen H, Chen H, Ming WK, Wang Z (2020). Incidence rate of type 2 diabetes mellitus after gestational diabetes mellitus: a systematic review and meta-analysis of 170,139 women. J Diabetes Res.

[10] Bao W, Yeung E, Tobias DK (2015). Long-term risk of type 2 diabetes mellitus in relation to BMI and weight change among women with a history of gestational diabetes mellitus: a prospective cohort study. Diabetologia.

[11] Blatt AJ, Nakamoto JM, Kaufman HW (2011). Gaps in diabetes screening during pregnancy and postpartum. Obstet Gynecol.

[12] Goueslard K, Cottenet J, Mariet AS, Sagot P, Petit JM, Quantin C (2017). Early screening for type 2 diabetes following gestational diabetes mellitus in France: hardly any impact of the 2010 guidelines. Acta Diabetol.

[13] Seidu S, Khunti K (2012). Non-adherence to diabetes guidelines in primary care - the enemy of evidence-based practice. Diabetes Res Clin Pract.

[14] Kim C, Newton KM, Knopp RH (2002). Gestational diabetes and the incidence of type 2 diabetes: a systematic review. Diabetes Care.

[15] Nouhjah S, Shahbazian H, Amoori N, Jahanfar S, Shahbazian N, Jahanshahi A, Cheraghian B (2017). Postpartum screening practices, progression to abnormal glucose tolerance and its related risk factors in Asian women with a known history of gestational diabetes: A systematic review and meta-analysis. Diabetes Metab Syndr.

[16] Bennewitz HG (1824). About Diabetes Mellitus, a Symptom of Pregnancy. Typis Ioannis Friderici Starckii.

[17] Świrska J, Zwolak A, Dudzińska M, Matyjaszek-Matuszek B, Paszkowski T (2018). Gestational diabetes mellitus - literature review on selected cytokines and hormones of confirmed or possible role in its pathogenesis. Ginekol Pol.

[18] Cukierman T, Gerstein HC, Williamson JD (2005). Cognitive decline and dementia in diabetes--systematic overview of prospective observational studies. Diabetologia.

[19] Begum S, Afroz R, Khanam Q, Khanom A, Choudhury T (2014). Diabetes mellitus and gestational diabetes mellitus. J Pediatr Surg Bangladesh.

[20] Stotland NE, Caughey AB, Breed EM, Escobar GJ (2004). Risk factors and obstetric complications associated with macrosomia. Int J Gynaecol Obstet.

[21] Ovesen PG, Jensen DM, Damm P, Rasmussen S, Kesmodel US (2015). Maternal and neonatal outcomes in pregnancies complicated by gestational diabetes. A nation-wide study. J Matern Fetal Neonatal Med.

[22] Ornoy A (2011). Prenatal origin of obesity and their complications: gestational diabetes, maternal overweight and the paradoxical effects of fetal growth restriction and macrosomia. Reprod Toxicol.

[23] Boney CM, Verma A, Tucker R, Vohr BR (2005). Metabolic syndrome in childhood: association with birth weight, maternal obesity, and gestational diabetes mellitus. Pediatrics.

[24] O'Sullivan JB (1991). Diabetes mellitus after GDM. Diabetes.

[25] Albareda M, Caballero A, Badell G, Piquer S, Ortiz A, de Leiva A, Corcoy R (2003). Diabetes and abnormal glucose tolerance in women with previous gestational diabetes. Diabetes Care.

[26] Vounzoulaki E, Khunti K, Abner SC, Tan BK, Davies MJ, Gillies CL (2020). Progression to type 2 diabetes in women with a known history of gestational diabetes: systematic review and meta-analysis. BMJ.

[27] Schaefer-Graf UM, Buchanan TA, Xiang AH, Peters RK, Kjos SL (2002). Clinical predictors for a high risk for the development of diabetes mellitus in the early puerperium in women with recent gestational diabetes mellitus. Am J Obstet Gynecol.

[28] Getahun D, Fassett MJ, Jacobsen SJ (2010). Gestational diabetes: risk of recurrence in subsequent pregnancies. Am J Obstet Gynecol.

[29] Lain KY, Catalano PM (2007). Metabolic changes in pregnancy. Clin Obstet Gynecol.

[30] Pratipanawatr W, Pratipanawatr T, Cusi K (2001). Skeletal muscle insulin resistance in normoglycemic subjects with a strong family history of type 2 diabetes is associated with decreased insulin-stimulated insulin receptor substrate-1 tyrosine phosphorylation. Diabetes.

[31] Reece EA, Leguizamón G, Wiznitzer A (2009). Gestational diabetes: the need for a common ground. Lancet.

[32] Metzger BE, Gabbe SG, Persson B (2010). The International Association of Diabetes and Pregnancy Study Groups recommendations on the diagnosis and classification of hyperglycemia in pregnancy. Diabetes Care.

[33] (2023). Definition, diagnosis and classification of diabetes mellitus and its complications: report of a WHO consultation. Part 1, Diagnosis and classification of diabetes mellitus. World.

[34] Defronzo RA (2009). Banting Lecture. From the triumvirate to the ominous octet: a new paradigm for the treatment of type 2 diabetes mellitus. Diabetes.

[35] Abdul-Ghani MA, Tripathy D, DeFronzo RA (2006). Contributions of beta-cell dysfunction and insulin resistance to the pathogenesis of impaired glucose tolerance and impaired fasting glucose. Diabetes Care.

[36] Gerstein HC, Santaguida P, Raina P (2007). Annual incidence and relative risk of diabetes in people with various categories of dysglycemia: a systematic overview and meta-analysis of prospective studies. Diabetes Res Clin Pract.

[37] Stumvoll M, Goldstein BJ, van Haeften TW (2005). Type 2 diabetes: principles of pathogenesis and therapy. Lancet.

[38] Cerf ME (2013). Beta cell dysfunction and insulin resistance. Front Endocrinol (Lausanne).

[39] Zheng Y, Ley SH, Hu FB (2018). Global aetiology and epidemiology of type 2 diabetes mellitus and its complications. Nat Rev Endocrinol.

[40] Harris MI, Klein R, Welborn TA, Knuiman MW (1992). Onset of NIDDM occurs at least 4-7 yr before clinical diagnosis. Diabetes Care.

[41] Barr RG, Nathan DM, Meigs JB, Singer DE (2002). Tests of glycemia for the diagnosis of type 2 diabetes mellitus. Ann Intern Med.

[42] (1997). Report of the Expert Committee on the Diagnosis and Classification of Diabetes Mellitus. Diabetes Care.

[43] Rushforth NB, Miller M, Bennett PH (1979). Fasting and two-hour post-load glucose levels for the diagnosis of diabetes. The relationship between glucose levels and complications of diabetes in the Pima Indians. Diabetologia.

[44] Sacks DB, Bruns DE, Goldstein DE, Maclaren NK, McDonald JM, Parrott M (2002). Guidelines and recommendations for laboratory analysis in the diagnosis and management of diabetes mellitus. Clin Chem.

[45] Saeedi P, Petersohn I, Salpea P (2019). Global and regional diabetes prevalence estimates for 2019 and projections for 2030 and 2045: Results from the International Diabetes Federation Diabetes Atlas, 9(th) edition. Diabetes Res Clin Pract.

[46] Lindahl B, Nilssön TK, Borch-Johnsen K (2009). A randomized lifestyle intervention with 5-year follow-up in subjects with impaired glucose tolerance: pronounced short-term impact but long-term adherence problems. Scand J Public Health.

[47] Tuomilehto J, Lindström J, Eriksson JG (2001). Prevention of type 2 diabetes mellitus by changes in lifestyle among subjects with impaired glucose tolerance. N Engl J Med.

[48] Ratner RE, Christophi CA, Metzger BE (2008). Prevention of diabetes in women with a history of gestational diabetes: effects of metformin and lifestyle interventions. J Clin Endocrinol Metab.

[49] Diaz-Santana MV, O'Brien KM, Park YM, Sandler DP, Weinberg CR (2022). Persistence of risk for type 2 diabetes after gestational diabetes mellitus. Diabetes Care.

[50] Chu SY, Callaghan WM, Kim SY, Schmid CH, Lau J, England LJ, Dietz PM (2007). Maternal obesity and risk of gestational diabetes mellitus. Diabetes Care.

[51] Lao TT, Ho LF, Chan BC, Leung WC (2006). Maternal age and prevalence of gestational diabetes mellitus. Diabetes Care.

[52] Jang HC (2011). Gestational diabetes in Korea: incidence and risk factors of diabetes in women with previous gestational diabetes. Diabetes Metab J.

[53] Buchanan TA, Xiang A, Kjos SL (1998). Gestational diabetes: antepartum characteristics that predict postpartum glucose intolerance and type 2 diabetes in Latino women. Diabetes.

[54] Metzger BE, Cho NH, Roston SM, Radvany R (1993). Prepregnancy weight and antepartum insulin secretion predict glucose tolerance five years after gestational diabetes mellitus. Diabetes Care.

[55] (2015). Standards of medical care in diabetes-2015 abridged for primary care providers. Clin Diabetes.

[56] Adekojo O, Revell KR, Preece H, Morris S, Coleman MA, Holt RI (2016). Low uptake of postpartum screening for type 2 diabetes in women after a diagnosis of gestational diabetes. Diabet Med.

[57] Song C, Lyu Y, Li C, Liu P, Li J, Ma RC, Yang X (2018). Long-term risk of diabetes in women at varying durations after gestational diabetes: a systematic review and meta-analysis with more than 2 million women. Obes Rev.

[58] Mahzari MM, Alwadi FA, Alhussain BM, Alenzi TM, Omair AA, Al Dera HS (2018). Development of type 2 diabetes mellitus after gestational diabetes in a cohort in KSA: prevalence and risk factors. J Taibah Univ Med Sci.

[59] Aziz S, Munim TF, Fatima SS (2018). Post-partum follow-up of women with gestational diabetes mellitus: effectiveness, determinants, and barriers. J Matern Fetal Neonatal Med.

[60] Chodick G, Elchalal U, Sella T, Heymann AD, Porath A, Kokia E, Shalev V (2010). The risk of overt diabetes mellitus among women with gestational diabetes: a population-based study. Diabet Med.

[61] Dennison RA, Chen ES, Green ME (2021). The absolute and relative risk of type 2 diabetes after gestational diabetes: a systematic review and meta-analysis of 129 studies. Diabetes Res Clin Pract.

[62] You H, Hu J, Liu Y, Luo B, Lei A (2021). Risk of type 2 diabetes mellitus after gestational diabetes mellitus: a systematic review &amp; meta-analysis. Indian J Med Res.

[63] Herath H, Herath R, Wickremasinghe R (2017). Gestational diabetes mellitus and risk of type 2 diabetes 10 years after the index pregnancy in Sri Lankan women-a community based retrospective cohort study. PLoS One.

[64] Huopio H, Hakkarainen H, Pääkkönen M, Kuulasmaa T, Voutilainen R, Heinonen S, Cederberg H (2014). Long-term changes in glucose metabolism after gestational diabetes: a double cohort study. BMC Pregnancy Childbirth.

[65] Retnakaran R, Qi Y, Sermer M, Connelly PW, Hanley AJ, Zinman B (2010). Beta-cell function declines within the first year postpartum in women with recent glucose intolerance in pregnancy. Diabetes Care.

[66] Wang Y, Chen L, Horswell R (2012). Racial differences in the association between gestational diabetes mellitus and risk of type 2 diabetes. J Womens Health (Larchmt).

[67] Mukerji G, Chiu M, Shah BR (2012). Impact of gestational diabetes on the risk of diabetes following pregnancy among Chinese and South Asian women. Diabetologia.

[68] Pintaudi B, Lucisano G, Pellegrini F (2015). The long-term effects of stillbirth on women with and without gestational diabetes: a population-based cohort study. Diabetologia.

[69] Vigneault J, Lemieux S, Garneau V, Weisnagel SJ, Tchernof A, Robitaille J (2015). Association between metabolic deteriorations and prior gestational diabetes according to weight status. Obesity (Silver Spring).

[70] Minooee S, Ramezani Tehrani F, Rahmati M, Mansournia MA, Azizi F (2017). Diabetes incidence and influencing factors in women with and without gestational diabetes mellitus: a 15 year population-based follow-up cohort study. Diabetes Res Clin Pract.

[71] Daly B, Toulis KA, Thomas N (2018). Increased risk of ischemic heart disease, hypertension, and type 2 diabetes in women with previous gestational diabetes mellitus, a target group in general practice for preventive interventions: a population-based cohort study. PLoS Med.

[72] Yefet E, Schwartz N, Sliman B, Ishay A, Nachum Z (2019). Good glycemic control of gestational diabetes mellitus is associated with the attenuation of future maternal cardiovascular risk: a retrospective cohort study. Cardiovasc Diabetol.

[73] England LJ, Dietz PM, Njoroge T, Callaghan WM, Bruce C, Buus RM, Williamson DF (2009). Preventing type 2 diabetes: public health implications for women with a history of gestational diabetes mellitus. Am J Obstet Gynecol.

[74] Nankervis A, McIntyre H, Moses R (2023). ADIPS consensus guidelines for the testing and diagnosis of hyperglycaemia in pregnancy in Australia and New Zealand. Australasian Diabetes in Pregnancy Society.

[75] Taylor JS, Kacmar JE, Nothnagle M, Lawrence RA (2005). A systematic review of the literature associating breastfeeding with type 2 diabetes and gestational diabetes. J Am Coll Nutr.

[76] Schwarz EB, Brown JS, Creasman JM, Stuebe A, McClure CK, Van Den Eeden SK, Thom D (2010). Lactation and maternal risk of type 2 diabetes: a population-based study. Am J Med.

[77] Jones SM, Wilson C (2010). Audit on outcome of midwife-led gestational diabetes care. Br J Midwifery.

[78] Liu J, Liu M, Chai Z (2023). Projected rapid growth in diabetes disease burden and economic burden in China: a spatio-temporal study from 2020 to 2030. Lancet Reg Health West Pac.

[79] Nicklas JM, Zera CA, Seely EW, Abdul-Rahim ZS, Rudloff ND, Levkoff SE (2011). Identifying postpartum intervention approaches to prevent type 2 diabetes in women with a history of gestational diabetes. BMC Pregnancy Childbirth.

[80] Moon JH, Kim H, Kim H (2020). Lactation improves pancreatic β cell mass and function through serotonin production. Sci Transl Med.

[81] Manerkar K, Harding J, Conlon C, McKinlay C (2020). Maternal gestational diabetes and infant feeding, nutrition and growth: a systematic review and meta-analysis. Br J Nutr.

[82] Nguyen PT, Pham NM, Chu KT, Van Duong D, Van Do D (2019). Gestational diabetes and breastfeeding outcomes: a systematic review. Asia Pac J Public Health.

[83] Nguyen PT, Binns CW, Nguyen CL (2019). Gestational diabetes mellitus reduces breastfeeding duration: a prospective cohort study. Breastfeed Med.

[84] Trout KK, Averbuch T, Barowski M (2011). Promoting breastfeeding among obese women and women with gestational diabetes mellitus. Curr Diab Rep.

[85] Gunderson EP, Hedderson MM, Chiang V (2012). Lactation intensity and postpartum maternal glucose tolerance and insulin resistance in women with recent GDM: the SWIFT cohort. Diabetes Care.

[86] Sterne V, Logan T, Palmer M (2011). Factors affecting attendance at postpartum diabetes screening in women with gestational diabetes mellitus. Pract Diabetes Int.

[87] Morrison MK, Collins CE, Lowe JM (2009). Postnatal testing for diabetes in Australian women following gestational diabetes mellitus. Aust N Z J Obstet Gynaecol.

[88] Baptiste-Roberts K, Barone BB, Gary TL, Golden SH, Wilson LM, Bass EB, Nicholson WK (2009). Risk factors for type 2 diabetes among women with gestational diabetes: a systematic review. Am J Med.

[89] McIntyre HD, Peacock A, Miller YD, Koh D, Marshall AL (2012). Pilot study of an individualised early postpartum intervention to increase physical activity in women with previous gestational diabetes. Int J Endocrinol.

[90] Clausen TD, Mathiesen ER, Hansen T (2009). Overweight and the metabolic syndrome in adult offspring of women with diet-treated gestational diabetes mellitus or type 1 diabetes. J Clin Endocrinol Metab.

[91] Metzger BE (2007). Long-term outcomes in mothers diagnosed with gestational diabetes mellitus and their offspring. Clin Obstet Gynecol.

[92] Lie ML, Hayes L, Lewis-Barned NJ, May C, White M, Bell R (2013). Preventing type 2 diabetes after gestational diabetes: women's experiences and implications for diabetes prevention interventions. Diabet Med.

[93] Adam S, McIntyre HD, Tsoi KY (2023). Pregnancy as an opportunity to prevent type 2 diabetes mellitus: FIGO Best Practice Advice. Int J Gynaecol Obstet.

[94] (2018). ACOG Practice Bulletin No. 190: gestational diabetes mellitus. Obstet Gynecol.

[95] (2021). 14. Management of diabetes in pregnancy: standards of medical care in diabetes-2021. Diabetes Care.

[96] Mukherjee SM, Dawson A (2022). Diabetes: how to manage gestational diabetes mellitus. Drugs Context.

[97] (2019). 14. Management of diabetes in pregnancy: standards of medical care in diabetes-2019. Diabetes Care.

[98] Vince K, Perković P, Matijević R (2020). What is known and what remains unresolved regarding gestational diabetes mellitus (GDM). J Perinat Med.

[99] Marín-Peñalver JJ, Martín-Timón I, Sevillano-Collantes C, Del Cañizo-Gómez FJ (2016). Update on the treatment of type 2 diabetes mellitus. World J Diabetes.

